# Factors contributing to sleep disturbances and excessive daytime sleepiness in patients with Parkinson's disease

**DOI:** 10.3389/fneur.2023.1097251

**Published:** 2023-03-09

**Authors:** Keitaro Ogaki, Hiroaki Fujita, Narihiro Nozawa, Tomohiko Shiina, Hirotaka Sakuramoto, Keisuke Suzuki

**Affiliations:** Department of Neurology, Dokkyo Medical University, Tochigi, Japan

**Keywords:** Parkinson's disease, sleep disturbances, excessive daytime sleepiness, rapid eye movement sleep behavior disorder, depressive symptoms, autonomic symptoms

## Abstract

**Background:**

Sleep disturbances and excessive daytime sleepiness (EDS) are common non-motor symptoms in patients with Parkinson's disease (PD). The purpose of this study was to identify the contributors to sleep disturbances, including insomnia, restless legs syndrome, rapid eye movement sleep behavior disorder (RBD), sleep-disordered breathing, nocturnal akinesia and EDS, in patients with PD.

**Methods:**

We conducted a cross-sectional study including 128 consecutive Japanese patients with PD. Sleep disturbances and EDS were defined as a PD Sleep Scale-2 (PDSS-2) total score ≥15 and an Epworth Sleepiness Scale (ESS) score >10, respectively. The patients were divided into four groups according to the presence or absence of sleep disturbances and EDS. We evaluated the disease severity, motor symptoms, cognition, olfactory test, the Scales for Outcomes in PD-Autonomic dysfunction (SCOPA-AUT), the Beck Depression Inventory-II (BDI-II), and the RBD Screening Questionnaire Japanese version (RBDSQ-J).

**Results:**

Of 128 patients, 64 had neither EDS nor sleep disturbances, 29 had sleep disturbances without EDS, 14 had EDS without sleep disturbances, and 21 had both EDS and sleep disturbances. Patients with sleep disturbances had higher BDI-II scores than those without sleep disturbances. Probable RBD was more frequent in patients with both sleep disturbances and EDS than in those with neither EDS nor sleep disturbances. The SCOPA-AUT score was lower in patients with neither EDS nor sleep disturbances than in patients in the other three groups. Using multivariable logistic regression analysis with neither sleep disturbances nor EDS as a reference group, that the SCOPA-AUT score was an independent contributor to sleep disturbances (adjusted OR, 1.192; 95% CI, 1.065–1.333; *P* = 0.002) or EDS (OR, 1.245; 95% CI, 1.087–1.424; *P* = 0.001) and that the BDI-II (OR, 1.121; 95% CI, 1.021–1.230; *P* = 0.016) and RBDSQ-J scores (OR, 1.235; 95% CI, 1.007–1.516; *P* = 0.043) as well as the SCOPA-AUT score (OR, 1.137; 95% CI, 1.006–1.285; *P* = 0.040) were independent contributors to both sleep disturbances and EDS.

**Conclusions:**

Autonomic symptoms were associated with patients with sleep disturbances or EDS, and depressive and RBD symptoms in addition to autonomic symptoms were associated with patients with both sleep disturbances and EDS.

## Introduction

Parkinson's disease (PD) is the most common neurodegenerative movement disorder (1) and is associated with Lewy body pathology in the central and peripheral nervous systems. Although PD is characterized by motor symptoms, such as bradykinesia, resting tremor, and rigidity, numerous non-motor symptoms appear, which often precede motor symptoms. Sleep disturbance is one of the major non-motor symptoms observed in 74–98% of individuals diagnosed with PD (2) and affects their overall quality of life (3). Sleep disturbances are typically multifactorial and may be influenced by the disease-related degeneration of wake-active monoaminergic and cholinergic neurons in the hypothalamus and brainstem, nocturnal motor and non-motor PD symptoms, coexisting sleep disorders such as rapid eye movement sleep behavior disorder (RBD), restless legs syndrome (RLS), obstructive sleep apnea (OSA) and circadian rhythm disorders, medications for PD and complications, and aging (4). Previous reports have suggested that some clinical conditions, including mood disorders (5) and autonomic symptoms (6), may have a close relationship with sleep disturbances and excessive daytime sleepiness (EDS). However, the clinical parameters that are significantly relevant to sleep conditions have not been fully elucidated. We speculate that the pathogenesis of sleep disturbances, which is subjective sleep assessment reported by patients, and EDS depends on the following three conditions: (1) the presence of both sleep disorders and EDS, (2) the presence of EDS but no sleep disorders, and (3) other factors (e.g., medication effects). The purpose of this study was to identify the contributors to sleep disturbances such as insomnia, RLS, RBD, OSA, nocturnal akinesia, and EDS in patients with PD.

## Methods

This single-center, cross-sectional study was performed in accordance with the Declaration of Helsinki guidelines and approved by the Institutional Review Board of Dokkyo Medical University. All participants provided written informed consent.

### Subjects

One hundred and twenty-eight consecutive Japanese patients with PD who visited the Department of Neurology, Dokkyo Medical University, between April 2018 and March 2022 were included. Bedridden patients who were unable to answer the questionnaire or who regularly used hypnotics or other sleep-promoting medications (such as antidepressants) were excluded from this study. Information on medical history that could potentially influence sleep conditions was collected from medical records. The diagnosis of PD was made based on the International Parkinson and Movement Disorder Society's published clinical diagnostic criteria (7).

### Clinical assessments

In the baseline assessment, disease severity was rated according to the Hoehn and Yahr (HY) stage (8). Motor symptoms were assessed by the Movement Disorder Society Unified Parkinson's Disease Rating Scale (MDS-UPDRS) part III. Cognitive function was assessed by the Japanese version of the Mini-Mental State Examination (MMSE) (9). Levodopa equivalent doses (LEDs) were calculated based on previously described methods (10). The percentage of dopamine agonist users in total was calculated. Olfactory function was examined by Open essence (Wako, Japan), a card-type odor identification test (11). Daytime sleepiness was evaluated by the Japanese version of the Epworth Sleepiness Scale (ESS) (12). PD-related sleep symptoms were assessed by the Japanese version of the Parkinson's Disease Sleep Scale-2 (PDSS-2), which consists of 15 PD-related sleep problems, including insomnia (questions 1–3), restless legs syndrome (questions 4–5), distressing dreams or hallucinations (questions 6–7), nocturia (question 8), nocturnal motor and non-motor symptoms (questions 9–13), sleepiness and fatigue (question 14), and sleep breathing problems (question 15) (13). Probable RBD was defined when patients had a history of dream-enacting behavior and scored 5 points or greater on the Japanese version of the RBD Screening Questionnaire (RBDSQ-J) (14). RLS was diagnosed through structured interviews based on 4 essential clinical features: the urge to move the legs, worsening of symptoms when at rest and during the night and relief with movement. RLS mimics were carefully excluded by neurological examination. Autonomic symptoms were assessed using the Japanese version of the Scales for Outcomes in Parkinson's Disease-Autonomic dysfunction (SCOPA-AUT) (15). The SCOPA-AUT consists of 25 subitems concerning autonomic symptoms related to PD, which are divided into 7 domains (gastrointestinal: items 1–7; urinary: items 8–13; cardiovascular: items 14–16; thermo: items 17, 18, 20, 21; pupillomotor: item 19; sexual function (male): items 22, 23; and sexual function (female): items 24, 25). Depressive symptoms were assessed with the Beck Depression Inventory-II (BDI-II).

Cardiac metaiodobenzylguanidine (MIBG) scintigraphy was included in the Movement Disorder Society's supportive criteria for PD reflecting postganglionic cardiac sympathetic denervation (7). The subjects were instructed to remain in a supine position for 15 min while 111 MBq 123I-MIBG (Fujifilm RI Pharma CO., Tokyo, Japan) was intravenously injected. Chest single photon emission computed tomography and planer images were obtained using a gamma camera 15 min (early phase) and 4 h (delayed phase) after the injection. The heart-to-mediastinum ratio (H/M ratio) was calculated by dividing the count density of the left ventricular region of interest (ROI) by that of the mediastinal ROI, as described previously (16). In the present study, H/M at the delayed phase was used.

In this study, patients with sleep disturbances and patients with EDS were defined as having a PDSS-2 total score ≥15 (17) and an ESS score >10 (18), respectively, based on previous reports. We divided the patients into four groups according to the presence or absence of sleep disturbances and EDS. We first compared the clinical parameters in the four groups and then evaluated the association between the clinical parameters and PDSS-2 or ESS scores separately. Finally, contributors to sleep disturbances and EDS were examined, adjusting for the effects of other confounding factors.

### Statistical analyses

We divided the patients into 4 groups: (1) patients with neither sleep disturbances nor EDS, (2) patients with sleep disturbances but no EDS, (3) patients with EDS but no sleep disturbances, and (4) patients with both sleep disturbances and EDS. To assess whether there was an overall difference, the Kruskal–Wallis test was used as appropriate to compare continuous variables. Similarly, the chi-square test was used to compare categorical variables. In *post-hoc* multiple comparisons, the Mann–Whitney *U*-test with Bonferroni correction was conducted. Spearman rank correlation coefficients were used to assess univariate correlations between the PDSS-2 or ESS scores and clinical variables. We performed a subgroup analysis of patients on PD treatment to assess the correlation between LEDs and the PDSS-2 and ESS scores. The multivariable logistic regression model with patients with neither sleep disturbances nor EDS as a reference group was performed to control for the potentially confounding factors for patients with sleep disturbances and EDS. As independent variables, age, sex, disease duration, MDS-UPDRS part III, BDI-II, RBDSQ-J, and SCOPA-AUT scores were included according to previous reports (5, 18–24). As a subgroup analysis, we performed a multivariable logistic regression analysis of *de novo* patients only.

## Results

The clinical characteristics of the patients with PD at baseline are presented in Table 1. The mean age, sex distribution, disease duration, percentage of *de novo* PD patients, HY stage, MDS-UPDRS Part III score, MMSE score, olfactory test results, MIBG cardiac scintigraphy results and LED did not significantly differ between the groups. The disease duration seemed to be shorter in patients with neither sleep disturbances nor EDS than in patients in the other three groups, but the difference was not significant. Similarly, the MIBG cardiac scintigraphy results and olfactory scores tended to be lower in the patients with EDS, but the difference was not significant. The BDI-II, RBDSQ-J, and SCOPA-AUT scores and the percentage of patients with RLS showed significant differences among the groups. The BDI-II scores were higher in patients with sleep disturbances than in those without sleep disturbances, but they did not differ in those with or without EDS. Probable RBD was more frequently observed in patients with both EDS and sleep disturbances compared with those with neither EDS nor sleep disturbances, but there was no significant difference between patients with only either EDS or sleep disturbances and those with neither EDS nor sleep disturbances. The SCOPA-AUT score was lower in patients with neither EDS nor sleep disturbances than in patients in the other three groups. The percentage of dopamine agonist users tended to be higher in patients with only EDS than in patients with neither EDS nor sleep disturbances.

**Table 1:** Comparison between patients with and without EDS and sleep disturbances (*n* = 128).

	**EDS-SD-**	**EDS-SD+**	**EDS+SD-**	**EDS+SD+**	**Kruskal–Wallis H**	***P-*** **value**
Patients, *n* (male/female)	64 (24/40)	29 (17/12)	14 (7/7)	21 (13/8)	5.739	0.123
Age (years)	68.7 ± 8.8	69.7 ± 9.4	68.8 ± 9.3	70.6 ± 7.0	1.247	0.742
Disease duration (years)	2.6 ± 2.5	4.1 ± 5.2	5.8 ± 4.4	4.4 ± 3.8	7.749	0.051
*De novo, n* (%)	33 (51.6%)	9 (31.0%)	5 (35.7%)	10 (47.6%)	3.910	0.268
HY stage, median value (interquartile range)	2.5(2.0–3.0)	3.0 (2.0–3.0)	2.0 (2.0–3.0)	3.0 (2.0–4.0)	7.344	0.108
MDS-UPDRS part III	26.9 ± 14.7	34.4 ± 16.6	24.1 ± 13.2	36.1 ± 18.5	7.150	0.067
MMSE	26.6 ± 2.9	26.3 ± 3.1	26.9 ± 3.1	24.7 ± 4.1	3.256	0.354
BDI-II	9.8 ± 6.8^a^^c^	15.0 ± 8.0	11.3 ± 8.6^c^	20.4 ± 10.7	25.366	**< 0.001**
PDSS-2	7.6 ± 3.6^a^^c^	22.2 ± 6.5^b^	8.7 ± 3.4^c^	21.9 ± 5.0	91.260	**< 0.001**
ESS	4.7 ± 2.8^b^^c^	4.7 ± 2.7^b^^c^	16.4 ± 5.3	16.1 ± 4.2	76.071	**< 0.001**
RBDSQ-J	1.9 ± 2.5^c^	2.6 ± 3.4	2.2 ± 3.7	4.5 ± 3.8	9.823	**0.020**
Probable RBD, *n* (%)	7(11%)^f^	7(24%)	2(14%)	7(33%)	10.829	**0.012**
SCOPA-AUT	7.7 ± 5.0^a^^b^^c^	14.5 ± 8.7	14.6 ± 8.7	15.5 ± 5.1	36.331	**< 0.001**
Olfactory test	4.1 ± 2.1	3.9 ± 2.6	3.4 ± 2.1	2.6 ± 1.9	7.718	0.052
MIBG cardiac scintigraphy H/M ratio (delayed phase)	2.0 ± 0.9	2.9 ± 4.6	1.5 ± 0.3	1.7 ± 0.7	3.531	0.317
LED (mg/day)	189.1 ± 256.1	249.8 ± 338.9	351.3 ± 374.8	313.4 ± 392.5	2.484	0.478
Use of dopamine agonists (%)	11 (17.2%)^e^	7 (24.1%)	7 (50.0%)	5 (23.8%)	6.854	0.075
RLS (%)	1 (1.6%)^d^^e^	6 (20.7%)	3 (21.4%)	2 (9.5%)	11.132	**0.011**

Statistically significant values (P < 0.05) before Bonferroni correction are shown in bold.

a Mann–ann–Whi U-tests, Bonferroni-corrected, compared with patients with SD and without EDS.

b Mann–ann–Whi U-tests, Bonferroni-corrected, compared with patients with EDS and without SD.

c Mann–ann–Whi U-tests, Bonferroni-corrected, compared with patients with both EDS and SD.

d Chi-square test, Bonferroni-corrected, compared with patients with SD and without EDS.

e Chi square test, Bonferroni-corrected, compared with patients with EDS and without SD.

f Chi square test, Bonferroni-corrected, compared with patients with both EDS and SD.

EDS-SD-, EDS, and SD both negative group; EDS-SD+, SD only positive group; EDS+SD-, EDS only positive group; EDS+SD+, EDS and SD both positive group.

EDS, excessive daytime sleepiness; SD, sleep disturbance; HY, Hoehn and Yahr; MDS-UPDRS, Movement Disorder Society Unified.

Parkinson's Disease Rating Scale; MMSE, Mini-Mental State Examination; BDI-II, Beck Depression Inventory-II; PDSS-2, Parkinson's Disease Sleep Scale-2; ESS, Epworth Sleepiness Scale; RBDSQ-J, RBD Screening Questionnaire Japanese version; SCOPA-AUT, Scales for Outcomes in Parkinson's Disease-autonomic dysfunction; MIBG, metaiodobenzylguanidine; H/M ratio, heart-to-mediastinum ratio; LED, levodopa equivalent dose; RLS, restless legs syndrome.

Data are presented as the n (%) or the mean ± SD.

Dopamine agonists included pramipexole (mean 1.35 mg, range 0.25–3.0 mg, *P* = 0.302) in 9 patients, ropinirole (mean 6.71 mg, range 4.0–12.0 mg, *P* = 0.079) in 7 patients, pergolide (0.375 mg) in 1 patient, cabergoline (mean 0.175 mg, range 1.0–3.0 mg, *P* = 0.368) in 3 patients and the rotigotine patch (mean 20.9 mg, range 4.5–36.0 mg, *P* = 0.309) in 10 patients, with no significant differences in usage among the four groups. RLS was more prevalent in patients with either EDS or sleep disturbances than in those with neither EDS nor sleep disturbances. Regarding comorbid diseases, 10 patients had cardiovascular disease (*P* = 0.911), 20 had diabetes mellitus (*P* = 0.478), 39 had hypertension (*P* = 0.336), 26 had dyslipidemia (*P* = 0.288), 6 had thyroid disease (*P* = 0.646), and 3 had rheumatoid arthritis (*P* = 0.117), with no significant differences among the four groups according to the Kruskal–Wallis test.

In the univariate correlation analysis, the PDSS-2 score was positively correlated with disease duration, HY stage, and the MDS-UPDRS part III, BDI-II, ESS, RBDSQ-J, and SCOPA-AUT scores and negatively correlated with the olfactory test results (Table 2). The ESS score was positively correlated with disease duration and the BDI-II, PDSS-2, RBDSQ-J and SCOPA-AUT scores (Table 3). The PDSS-2 and ESS scores showed a positive correlation with all domains of the SCOPA-AUT score except for sexual function (data not shown). A subgroup analysis of patients on PD treatment showed that there was no correlation between LEDs and the PDSS-2 or ESS score.

**Table 2:** Univariate correlation coefficients between PDSS-2 scores and clinical parameters.

**Correlation coefficient (** ***r*** **)**	
Age (years)	0.101
Disease duration	**0.182** ^*^
HY stage	**0.189** ^*^
MDS-UPDRS part III	**0.258** ^**^
MMSE	−0.164
BDI-II	**0.427** ^**^
ESS	**0.271** ^**^
RBDSQ-J	**0.313** ^**^
SCOPA-AUT	**0.495** ^**^
Olfactory test	**−0.203** ^*^
MIBG cardiac scintigraphy H/M ratio (delayed phase)	**–**0.029
LED	0.114

PDSS-2, Parkinson's Disease Sleep Scale-2; HY, Hoehn and Yahr; MDS-UPDRS, Movement Disorder Society Unified Parkinson's Disease Rating Scale; MMSE, Mini-Mental State Examination; BDI-II, Beck Depression Inventory-II; ESS, Epworth Sleepiness Scale; RBDSQ-J, RBD Screening Questionnaire Japanese version; SCOPA-AUT, Scales for Outcomes in Parkinson's Disease-autonomic dysfunction; MIBG, metaiodobenzylguanidine; H/M ratio, heart-to-mediastinum ratio; LED, levodopa equivalent dose.

* *p* < 0.05.

** *p* < 0.01.

Statistically significant values (*p* < 0.05) are shown in bold.

**Table 3:** Univariate correlation coefficients between ESS and clinical parameters.

**Correlation coefficient (** ***r*** **)**	
Age (years)	−0.068
Disease duration	**0.284** ^**^
HY stage	0.126
MDS-UPDRS part III	0.076
MMSE	−0.094
BDI-II	**0.304** ^**^
PDSS-2	**0.271** ^**^
RBDSQ-J	**0.257** ^**^
SCOPA-AUT	**0.412** ^**^
Olfactory test	−0.127
MIBG cardiac scintigraphy H/M ratio (delayed phase)	−0.159
LED	0.135

ESS, Epworth Sleepiness Scale; HY, Hoehn and Yahr; MDS-UPDRS, Movement Disorder Society Unified Parkinson's Disease Rating Scale; MMSE, Mini-Mental State Examination; BDI-II, Beck Depression Inventory-II; PDSS-2, Parkinson's Disease Sleep Scale-2; RBDSQ-J, RBD Screening Questionnaire Japanese version; SCOPA-AUT, Scales for Outcomes in Parkinson's Disease-Autonomic dysfunction; MIBG, metaiodobenzylguanidine; H/M ratio, heart-to-mediastinum ratio; LED, levodopa equivalent dose.

** *p* < 0.01.

Statistically significant values (p < 0.05) are shown in bold.

Multivariable logistic regression analysis of patients with neither sleep disturbances nor EDS as a reference group revealed that the SCOPA-AUT score was an independent contributor in patients with sleep disturbances [adjusted odds ratio (OR), 1.192; 95% confidence interval (CI), 1.065–1.333; *P* = 0.002] or EDS (OR, 1.245; 95% CI, 1.087–1.424; *P* = 0.001) and that the BDI-II (OR, 1.121; 95% CI, 1.021–1.230; *P* = 0.016) and RBDSQ-J scores (OR, 1.235; 95% CI, 1.007–1.516; *P* = 0.043) as well as the SCOPA-AUT score (OR, 1.137; 95% CI, 1.006–1.285; *P* = 0.040) were independent contributors in patients with both sleep disturbances and EDS (Table 4). In the subgroup analysis of untreated patients (*n* = 57), the SCOPA-AUT score (OR, 1.281; 95% CI, 1.037–1.582; *P* = 0.022) was an independent contributor in patients with only sleep disturbances, and the BDI-II (OR, 1.596; 95% CI, 1.091–2.334; *P* = 0.016) and SCOPA-AUT scores (OR, 1.598; 95% CI, 1.110–2.301; *P* = 0.012) were independent contributors in patients with sleep disturbances and EDS.

**Table 4:** Multivariable logistic regression analysis with EDS-SD- as a reference group.

**Variable**	**EDS-SD**+		**EDS**+**SD-**		**EDS**+**SD**+	
	**OR (95% CI)**	***P-*** **value**	**OR (95% CI)**	***P-*** **value**	**OR (95% CI)**	***P-*** **value**
Age	0.999 (0.932–1.071)	0.985	1.024 (0.940–1.115)	0.585	1.012 (0.933–1.098)	0.772
Sex	1.998 (0.619–6.449)	0.247	3.479 (0.806–15.012)	0.095	2.030 (0.538–7.660)	0.296
Disease duration	1.064 (0.900–1.257)	0.469	1.163 (0.984–1.375)	0.076	1.028 (0.845–1.249)	0.783
MDS-UPDRS Part III	1.021 (0.984–1.059)	0.271	0.965 (0.910–1.025)	0.246	1.026 (0.986–1.068)	0.207
BDI-II	1.054 (0.966–1.150)	0.237	0.968 (0.764–1.257)	0.582	1.121 (1.021–1.230)	**0.016**
SCOPA-AUT	1.192 (1.065–1.333)	**0.002**	1.245 (1.087–1.424)	**0.001**	1.137 (1.006–1.285)	**0.040**
RBDSQ-J	1.051 (0.858–1.286)	0.631	0.980 (0.764–1.257)	0.873	1.235 (1.007–1.516)	**0.043**

Statistically significant values (P < 0.05) are shown in bold.

EDS-SD-, EDS, and SD both negative group; EDS-SD+, SD only positive group; EDS+SD-, EDS only positive group; EDS+SD+, EDS and SD both positive group.

EDS, excessive daytime sleepiness; SD, sleep disturbance; PD, Parkinson's disease; BDI-II, Beck Depression Inventory-II; MDS-UPDRS, Movement Disorder Society Unified Parkinson's Disease Rating Scale; RBDSQ-J, RBD Screening Questionnaire Japanese version; SCOPA-AUT, Scales for Outcomes in Parkinson's Disease-autonomic dysfunction.

## Discussion

In the present study, patients with sleep disturbances had higher depressive symptom scores than those without sleep disturbances, regardless of the presence or absence of EDS. More patients with probable RBD were observed in patients with both EDS and sleep disturbances than in patients with neither EDS nor sleep disturbances, but there was no significant difference between patients with only EDS or sleep disturbances. On the other hand, the SCOPA-AUT score was lower in the group with neither EDS nor sleep disturbances, and there was no significant difference between the group with both EDS and sleep disturbances and the group with only EDS or sleep disturbances. Univariate correlation analysis showed a positive relationship between the PDSS-2 or ESS score and the BDI-II, SCOPA-AUT and RBDSQ-J scores. Multivariable logistic regression analysis adjusted for age, sex, disease duration and the MDS-UPDRS part III score revealed that the SCOPA-AUT score was an independent contributor in patients with sleep disturbances or EDS and that the SCOPA-AUT, BDI-II, and RBDSQ-J scores were independent contributors in patients with sleep disturbances and EDS. In the subgroup analysis of untreated patients only, the SCOPA-AUT and BDI-II scores showed similar associations with patients with sleep disturbances and EDS. Taken together, autonomic symptoms were associated with patients with sleep disturbances or EDS, and depressive and RBD symptoms in addition to autonomic symptoms were associated with patients with both sleep disturbances and EDS.

Previous studies have suggested that there is a reciprocal relationship between depression and sleep disturbances in patients with PD (25). Although the underlying mechanisms of depression in patients with PD have not been completely elucidated, dysfunction in some specific brain areas, such as the brainstem monoaminergic system, may be involved (26). It is postulated that dysfunction in cholinergic and monoaminergic neuron systems may be related to both depression and sleep disturbances in patients with PD, given that serotonin and norepinephrine reuptake inhibitors are effective for treating these symptoms, although depressive symptoms in patients with PD are influenced by complex clinical factors (25). Suzuki et al. (5) conducted a multicenter cross-sectional study and showed that nocturnal disturbances and the UPDRS part I (mental state) score were associated with depressive symptoms in patients with PD. Our results were consistent with this report, and depressive symptoms were contributors in patients with sleep disturbances and EDS independent of disease duration and motor symptoms.

We previously reported that compared to those without probable RBD, patients with PD and probable RBD diagnosed with RBDSQ-J showed severe autonomic and sleep-related symptoms, including visual hallucinations and sleepiness (19). Previous studies also indicated the correlation between probable RBD diagnosed with RBDSQ and sleep disturbances or sleepiness in patients with PD (27, 28). The pathogenesis of RBD is considered to be based on a specific degeneration of REM-on glutamatergic neurons localized in the caudal pontine sublaterodorsal tegmental nucleus or the glycinergic/GABAergic premotor neurons localized in the medullary ventral gigantocellular reticular nucleus (29). Postuma et al. (30) conducted a pathological study and showed that patients with PD and probable RBD diagnosed through interview showed somewhat severe alpha-synuclein pathology in both cortical and subcortical regions. Recently, brain-first and body-first PD were proposed as new models of pathological propagation. Body-first PD, which is characterized as RBD positive at motor symptom onset, displays a large burden of autonomic symptoms, frequent alpha-synuclein deposition in peripheral tissues, and more brainstem involvement (31). In sum, patients with PD comorbid with RBD are associated with a severe subtype of neurodegenerative progression, mainly in the brainstem, which may result in severe sleep disturbances and EDS.

The present study suggested that autonomic symptoms were associated with both nocturnal sleep disturbances and daytime sleepiness in patients with PD. Recently, Zhou et al. (32) reported that the PDSS score was negatively correlated with the SCOPA-AUT score in a Chinese population, although a logistic regression model failed to indicate a correlation between them. Although the underlying mechanism of the relationship between autonomic symptoms and sleep disturbances or EDS is unknown, it is well described that abnormal aggregation of alpha-synuclein in the central and peripheral autonomic networks produces dysfunction and neurodegeneration in patients with synucleinopathies (33). In patients with PD, the peripheral autonomic system is typically more susceptible and may be the essential route for the propagation of alpha-synuclein pathology from the periphery to the central nervous system. Severe autonomic symptoms suggest intense disease-related neurodegeneration in the central and peripheral nervous systems and the sleep-wake system, which is maintained primarily by brainstem nuclei and may also be greatly disturbed. The data-driven clustering approaches (34) indicated that dysautonomia, RBD, and cognitive and motor symptoms are indicators determining clinical subtypes that predict subsequent PD outcome, although the impact of RBD diagnosed with the RBDSQ on the progression of motor and non-motor symptoms in PD was controversial in a recent study (35). Autonomic symptoms reflect a high degree of pathological changes in patients with PD and may be an indicator that is closely related to sleep disorders and EDS. In the present study, however, MIBG scintigraphy did not show a significant difference among the groups, although the score tended to be lower in patients with EDS than in those without EDS. An alternative possibility is that autonomic symptoms, such as nocturia and thermoregulation disorder, directly disrupt sleep maintenance and result in frequent arousals. The PDSS-2 and ESS scores showed a positive correlation with all domains of the SCOPA-AUT except for sexual function, suggesting a close correlation between sleep disruption and autonomic symptoms.

In a previous study, the use of dopamine agonists was correlated with EDS (21), although some previous studies indicated controversial results (22). In the present study, the LEDs did not show significant differences among the groups, although the percentage of dopamine agonist users in total tended to be higher in patients with only EDS than in patients with neither EDS nor sleep disturbances. We conducted a subgroup analysis of only patients receiving PD treatment, but no significant correlation was found between LEDs and the PDSS-2 or ESS score. Tholfsen et al. (36) showed that the prevalence of EDS was higher in patients with PD than in controls, even in early, drug-naïve patients. In the present study, nearly half of the patients were drug naïve, and the LEDs were relatively low, suggesting that the medications had less impact on the patients' sleep-related symptoms.

The strength of the present study was the high percentage of *de novo* patients. Among the 57 *de novo* patients, 19 (33%) had sleep disturbances, and 15 (26%) had EDS. Our results confirmed that a significant number of patients with PD experience sleep disturbances and EDS at an early stage, even though medication for PD has not yet been initiated. A subgroup analysis in this population confirmed that autonomic and depressive symptoms were independent contributors to sleep disturbances and EDS.

The present study has some limitations. First, we lacked a control group. However, we conducted a comprehensive detailed clinical assessment, including cardiac MIBG scintigraphy and olfactory testing. Second, we assessed nocturnal sleep, daytime sleepiness and the definition of prodromal RBD using questionnaires. Although polysomnography and multiple sleep latency tests are objective and have strong diagnostic power, it is not practical to perform these procedures in all patients with PD. Third, lack of temporal information regarding the onset of RBD, conducted cardiac MIBG scintigraphy, and onset of motor symptoms made it impossible to discuss the difference between body-first and brain-first types. Fourth, the cross-sectional design of this study did not allow for interpretation of the direction of causality. Follow-up studies are needed.

## Conclusions

In our study, we found that autonomic symptoms were independent contributors in PD patients with sleep disturbances or EDS and that RBD symptoms and depressive symptoms in addition to autonomic symptoms were independent contributors in PD patients with both sleep disturbances and EDS.

## Data availability statement

The original contributions presented in the study are included in the article/supplementary material, further inquiries can be directed to the corresponding author.

## Ethics statement

The studies involving human participants were reviewed and approved by Institutional Review Board of Dokkyo Medical University. The patients/participants provided their written informed consent to participate in this study.

## Author contributions

Conceptualization: HF, KO, TS, HS, NN, and KS. Data curation and writing—original draft: HF and KO. Formal analysis and supervision: KS. Investigation and methodology: TS and HS. Project administration: HF and NN. Writing—review and editing: TS, HS, and KS. All authors contributed to the article and approved the submitted version.
